# Serum leptin is associated with cardiometabolic risk and predicts metabolic syndrome in Taiwanese adults

**DOI:** 10.1186/1475-2840-10-36

**Published:** 2011-04-28

**Authors:** Wen-Cheng Li, Kuang-Yu Hsiao, I-Chuan Chen, Yu-Che Chang, Shih-Hao Wang, Kuan-Han Wu

**Affiliations:** 1 Department of Occupation Medicine, Chang-Gung Memorial Hospital, Keelung Branch, No. 222, Maijin Rd., Keelung, Taiwan; 2 Department of Emergency Medicine, Chang Gung Memorial Hospital, Chiayi Branch, No. 6, West sec. Chia-Pu Rd, Put-Zu, Chiayi, Taiwan; 3 Chang Gung Institute of Technology, Chiayi Branch, No. 2, West sec. Chia-Pu Rd, Put-Zu, Chiayi, Taiwan; 4 Department of Emergency Medicine, Chang-Gung Memorial Hospital, Linkou Branch, No.5, Fu-Hsing Street, Guei-Shan, Taoyuan, Taiwan; 5 College of Medicine, Chang-Gung University, No. 259, Wen-Hwa 1st Rd., Guei-Shen, Taoyuan, Taiwan; 6 Institute of Environmental and Occupational Health Science, National Yang-Ming University, Taipei, Taiwan; 7 Department of Emergency Medicine, Chang-Gung Memorial Hospital, Kaohsiung Branch, No.123, Dapi Rd., Niaosong Township, Kaohsiung County 833, Taiwan

**Keywords:** Taiwan, obesity, metabolic syndrome

## Abstract

**Background:**

Leptin is associated with cardiovascular disease (CVD); however, few studies have assessed its relationship with metabolic syndrome, especially in an Asian population. Therefore, the aim of the present study was to assess leptin levels and evaluate its association with CVD and metabolic syndrome.

**Methods:**

In 2009, 957 subjects, who underwent a routine physical examination and choose leptin examination, were selected to participate. Participants (269 females and 688 males) were stratified according to leptin level quartiles. Metabolic syndrome was defined by NCEP ATP III using waist circumference cutoffs modified for Asian populations, and CVD risk was determined using the Framingham Heart Study profile.

**Results:**

Leptin levels were correlated with CVD risk in men and women. With the exception of fasting plasma glucose, increased leptin levels were observed as factors associated with metabolic syndrome increased in both males and females. After adjusting for age, an association between leptin levels and metabolic syndrome was observed. After adjusting for age alone or with tobacco use, subjects in the highest leptin quartile had a higher risk of having metabolic syndrome than those in the lowest quartile (OR = 6.14 and 2.94 for men and women, respectively). After further adjustment for BMI, metabolic syndrome risk remained significantly increased with increasing leptin quartiles in men. Finally, increased leptin levels were a predictor of metabolic syndrome in men and women.

**Conclusions:**

Serum leptin levels are correlated with CVD risk and metabolic syndrome. Analysis of leptin as part of routine physical examinations may prove beneficial for early diagnosis of metabolic syndrome.

## Introduction

Metabolic syndrome is a major risk factor for type 2 diabetes mellitus and cardiovascular disease (CVD). Specifically, a three-fold increase in the prevalence of metabolic syndrome is associated with a two-fold increased risk of cardiovascular disease fatality, 150% increase in total mortality, and a five-fold increased risk of diabetes mellitus [1]. Because metabolic syndrome is associated with increased risk for CVD and diabetes mellitus [2-5], early diagnosis of metabolic syndrome and resultant intervention strategies may help reduce the incidence of these associated diseases.

Leptin is important for body weight regulation. In mice, mutations of the *OB *gene on chromosome 7, which encodes the leptin protein, results in obesity and type 2 diabetes [6]. The amount of leptin secreted by white adipose tissue is proportional to the volume of body adipose tissue; therefore, adiposity greatly influences leptin levels. In addition, other factors, including rapid or excessive food intake, sleep, body temperature, gender, circadian rhythm, and other hormones such as insulin, growth hormone, glucocorticoids, testosterone, and thyroid hormone, impact leptin secretion and expression.

Elevated serum leptin concentration is a feature of obesity and abdominal adiposity, a risk factor for metabolic syndrome. Although elevated serum leptin is not considered among the diagnostic criteria for metabolic syndrome, it is increased in subjects with metabolic syndrome [7-9].

Because leptin has a certain degree of influence on appetite, energy consumption, adipose synthesis, and insulin function, elevated serum leptin levels are common in human obesity. The higher the body mass index (BMI) or the waist circumference, the higher the serum leptin level [10]. The elevated concentration of circulating leptin has been consistently associated with other cardiometabolic risk factors, such as hypertension, insulin resistance, and type 2 diabetes [11-18]; however, few studies have analyzed its association with metabolic syndrome [7-9]. Of the few conducted, few have analyzed Asian populations. Thus, the objective of the present study was to assess the association of leptin with metabolic syndrome and CVD risk in Taiwanese adults as well as the predictive value of leptin levels for identifying patients with metabolic syndrome. Leptin levels were measured in adult participants undergoing a routine physical examination, and their relationship with metabolic syndrome as well as CVD risk was assessed.

## Materials and methods

### Study participants

In 2009, 988 subjects > 18 years of age, who underwent a routine physical examination and choose leptin examination in Chang-Gung Memorial Hospital-Linkou (North Taiwan) and Chiayi (South Taiwan), were selected to participate in this retrospective study. Questionnaires were completed at the Health Examination Centers in the Linkou and Chiayi branches of Cheng-Gung Memorial Hospital; the physical examination and venous blood sampling was obtained at the same time. Analysis excluded subjects that did not fast for more than 10 h prior to blood sampling, were pregnant, those with chronic diseases that significantly impact the metabolic functions, such as thyroid diseases, chronic hepatitis, liver cirrhosis, hypothalamus disease, etc., and those with a history of illness or medication use in the questionnaires. There were 31 subjects excluded from this study based on the exclusion criteria. Therefore, a total of 957 participants (688 males, average age of 35.2 y [SD = 7.2 y] and 269 females, average age of 38.5 y [SD = 11.8 y]) were included for this study. The study protocol was approved by the Institutional Review Board of Chang-Gung Memorial Hospital.

### Clinical and biochemical measurements

Participants were interviewed using questionnaires by trained nurses. Information regarding demographic (e.g., age, gender, etc.) and life style (e.g., experience of smoking, drinking, etc.) characteristics, history of illness and medication use, and physiologic status (pregnancy, fasting time, etc.) was collected.

Blood pressure was measured after the participants were at rest for 10 min using an automatic sphygmomanometer to the right arm while the individuals were in a sitting position. An additional 5-minute break was given between measurements for participants with systolic blood pressure > 140 mmHg or the diastolic blood pressure > 90 mmHg. The lowest value was used after 2 to 3 measurements. The participant's weight and height was measured without shoes and after fasting. BMI was calculated as weight (kg) divided by the square of height (m^2^). Waist circumference was measured midway between the iliac crest and the lower margin of the 12^th ^rib.

After a 12-h fast, venous blood samples were obtained from 5:30 to 11:00 a.m. and stored at 4°C. Total cholesterol, high-density lipoprotein cholesterol (HDL-C), triglyceride (TG), fasting blood glucose (FBG), low-density lipoprotein cholesterol (LDL-C), uric acid, homocysteine, and leptin levels were determined for each participant.

### Definition of metabolic syndrome

A diagnosis of metabolic syndrome was defined as a subject presenting at least 3 of the 5 factors for metabolic syndrome described by the Third Adult Treatment Panel (ATP III) of the National Cholesterol Education Program (NCEP) [19]. The diagnostic criteria were defined as follows: 1) high blood pressure (a systolic blood pressure ≥ 130 mmHg and/or diastolic pressure ≥ 85 mmHg, under treatment, or already diagnosed with hypertension); 2) high serum triglyceride (≥ 150 mg/dL or under treatment); 3) decreased HDL-C (< 40 mg/dL for males and < 50 mg/dL for females or under treatment); 4) hyperglycemia (FBG ≥ 100 mg/dL, under treatment, or previously diagnosed with diabetes mellitus); and abdominal obesity. Waist circumference cutoffs were modified for Asian populations [20]. A waist circumference ≥ 90 cm for men and ≥ 80 cm for women plus the other two risk factors or the waist circumstance within the threshold plus the other three or more risk factors resulted in a diagnosis of metabolic syndrome.

### Cardiovascular disease risk percentage definition

CVD risk was analyzed based upon the profile described by D'Agostino et al. [21]. This profile predicts the risk of developing all CVD events, including coronary death, myocardial infarction, coronary insufficiency, angina, ischemic stroke, hemorrhagic stroke, transient ischemic attack, peripheral artery disease, heart failure, using sex-specific multivariable risk functions that incorporated age, total cholesterol, HDL-C, systolic blood pressure, treatment for hypertension, smoking, and diabetes status.

### Statistical analysis

Serum leptin levels were categorized into quartiles (Q1-4) according to the leptin levels (Q1, < 2.4 ng/mL; Q2, 2.4 to < 4.2 ng/mL; Q3, 4.2 to < 6.65 ng/mL; and Q4 ≥ 6.65 ng/mL for males and Q1, < 6.8 ng/mL; Q2, 6.8 to <9.0 ng/mL; Q3, 9.0 to < 13.1 ng/mL; and Q4, ≥ 13.1 ng/mL for females). The data were presented as mean ± standard deviation (SD) for continuous variables and n (%) for categorical variables. Clinical characteristics were compared using one-way ANOVA for continuous variables and Chi-sqaure or Fisher's exact test for categorical variables. Spearman's rank correlation was performed for each gender to assess the relationship between leptin levels and cardiometabolic risk factors. General linear model analysis was applied to identify the association of leptin levels with cardiometabolic risk factors after adjusting for age and tobacco usage. In addition, a binary logistic regression model was used to evaluate the association of leptin levels with metabolic syndrome after adjusting for age, tobacco usage, and BMI. A received operating curve (ROC) for leptin level as a predictor of metabolic syndrome was also carried out. Statistical analyses were performed using the SPSS 15.0 software package (SPSS, Inc., Chicago, IL). A *P*-value < 0.05 was considered statistically significant.

## Results

The characteristics of male and female participants stratified by leptin quartiles are shown in Table 1. The mean leptin level for the study participants was 1.42 ng/mL. The prevalence of metabolic syndrome was 61% in the study population. In men and women, participants in Q4 were more likely to have factors associated with metabolic syndrome, including increased waist circumference, triglyceride, and blood pressure and decreased HDL-C. No significant differences in FBG were observed. In addition, no differences in tobacco usage and homocysteine level were observed (Table 1). Furthermore, those with metabolic syndrome were more likely to be in the upper leptin quartiles. Finally, percentage of CVD risk significantly increased with increasing leptin levels in both men and women.

**Table 1:** General characteristics of the study population according to sex-specific leptin levels.

	Leptin levels				
		
Characteristics	Q1	Q2	Q3	Q4	***P*-value**^**c**^
**Men**	**(n = 149)**	**(n = 186)**	**(n = 181)**	**(n = 171)**	
Age^a^, y	34.9 ± 7.7	34.5 ± 6.8	36.5 ± 8.6	34.7 ± 5.3	0.041*
Body mass index^a^, kg/m^2^	21.5 ± 2.3	23.6 ± 2	25.7 ± 2.5	27.8 ± 3.2	< 0.001*
Waist circumference^a^, cm	76.1 ± 5.9	82.2 ± 5	87.9 ± 6.9	92.7 ± 7.7	<0.001*
Tobacco usage^b^, n (%)	65 (43.6)	76 (40.9)	77 (42.5)	75 (43.6)	0.947
Systolic blood pressure^a^, mm Hg	119.6 ± 9.7	122 ± 11.2	125.9 ± 11.9	127.7 ± 13.7	< 0.001*
Diastolic blood pressure^a^, mm Hg	74.7 ± 7.1	76.3 ± 8.2	78.4 ± 7.5	81.3 ± 9.9	< 0.001*
Total cholesterol^a^, mg/dL	179.5 ± 29.5	189.4 ± 28.6	192.2 ± 31	198.6 ± 36.8	< 0.001*
Triglyceride^a^, mg/dL	91.2 ± 44.4	128.2 ± 114.6	144.3 ± 83.5	153.8 ± 87	< 0.001*
Fasting plasma glucose^a^, mg/dL	89.1 ± 19.2	90.5 ± 19.5	92.1 ± 14.8	93.2 ± 13.4	0.135
Uric acid^a^, mg/dL	6.2 ± 1	6.7 ± 1.3	6.9 ± 1.2	7.2 ± 1.3	< 0.001*
HDL cholesterol^a^, mg/dL	56.6 ± 13.2	51.2 ± 11.2	47.5 ± 9.2	46.9 ± 9.2	< 0.001*
LDL cholesterol^a^, mg/dL	115.5 ± 27.5	126.1 ± 26.2	131 ± 29.3	137.6 ± 33.3	< 0.001*
Homocysteine^a^, umol/L	10.5 ± 3	10.2 ± 3.3	10.1 ± 3.3	10.8 ± 5.7	0.338
Leptin^a^, ng/mL	1.4 ± 0.6	3.2 ± 0.5	5.2 ± 0.7	10.3 ± 4.7	< 0.001*
Metabolic syndrome^b^, n (%)	60 (40.3)	125 (67.2)	141 (77.9)	137 (79.7)	< 0.001*
Percentage of CVD risk^a^	2.47 ± 4.14	3.49 ± 4.36	5.03 ± 4.69	4.91 ± 4.02	< 0.001*
**Women**	**(n = 65)**	**(n = 67)**	**(n = 68)**	**(n = 69)**	
Age^a^, y	36.5 ± 11.3	37.7 ± 12	38.5 ± 10.9	41.3 ± 12.7	0.111
Body mass index^a^, kg/m^2^	19.8 ± 2	20.9 ± 1.9	22.1 ± 2.4	24.8 ± 3.6	<0.001*
Waist circumference^a^, cm	68.6 ± 7	69.6 ± 5.6	73 ± 7.3	79.4 ± 10.3	< 0.001*
Tobacco usage^b^, n (%)	4 (6.2)	3 (4.5)	2 (2.9)	2 (2.9)	0.717
Systolic blood pressure^a^, mm Hg	110.7 ± 13.4	110.2 ± 11.1	112.5 ± 13.8	118.2 ± 15.2	0.002*
Diastolic blood pressure^a^, mm Hg	69.9 ± 8.4	68.6 ± 6.9	70.7 ± 10.2	73.7 ± 7.2	0.004*
Total cholesterol^a^, mg/dL	180.4 ± 30.9	181 ± 33.3	187 ± 28.9	195.9 ± 33.3	0.016*
Triglyceride^a^, mg/dL	84.1 ± 60.4	85.1 ± 98.5	90.5 ± 42.8	123.9 ± 82.8	0.005*
Fasting plasma glucose^a^, mg/dL	87.7 ± 24.3	88.9 ± 12.8	87.5 ± 7.4	92.2 ± 17.7	0.325
Uric acid^a^, mg/dL	4.7 ± 0.8	4.6 ± 0.9	4.9 ± 1.1	5.3 ± 1.1	<0.001*
HDL cholesterol^a^, mg/dL	64.2 ± 14.2	63.6 ± 12.2	61.2 ± 13.9	57.6 ± 11.6	0.016*
LDL cholesterol^a^, mg/dL	107.8 ± 28.6	109.4 ± 28.9	117.5 ± 26.4	126.5 ± 31.7	0.001*
Homocysteine^a^, umol/L	7.8 ± 1.9	7.6 ± 1.7	7.6 ± 1.7	7.8 ± 1.4	0.727
Leptin^a^, ng/mL	4.8 ± 1.3	7.5 ± 0.6	10.9 ± 1.3	17.6 ± 3.9	< 0.001*
Metabolic syndrome^b^, n (%)	21 (32.3)	26 (38.8)	31 (45.6)	43 (62.3)	0.003*
Percentage of CVD risk^a^	-0.38 ± 4.14	0.19 ± 5.01	0.75 ± 5.41	2.72 ± 6.39	0.005*

Serum leptin levels were categorized into quartiles (Q1-4) according to the leptin levels (Q1, < 2.4 ng/mL; Q2, 2.4 to < 4.2 ng/mL; Q3, 4.2 to < 6.65 ng/mL; and Q4 ≥ 6.65 ng/mL for males and Q1, < 6.8 ng/mL; Q2, 6.8 to <9.0 ng/mL; Q3, 9.0 to < 13.1 ng/mL; and Q4, ≥ 13.1 ng/mL for females).

^a,b ^Clinical characteristics were presented as mean ± SD for ^a^continuous variables and n (%) for ^b^categorical variables.

^c ^*P*-values were derived through one-way ANOVA for continuous variables and Chi-square or Fisher's exact test for categorical variables.

**Figure 1:**
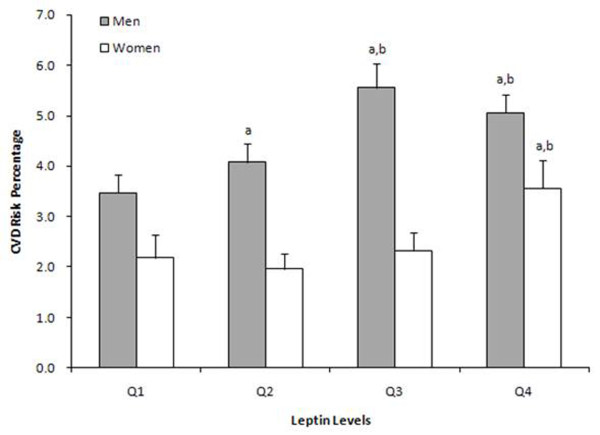
**The distribution of CVD risk percentage by sex-specific leptin levels**. Data represent mean with standard error of mean for each leptin quartile. ^a ^significantly different (P < 0.0167) in CVD risk % as compared with Q1. ^b ^significantly different (P < 0.0167) in CVD risk % as compared with Q2.

The relationship between increased leptin levels and CVD risk percentage was also analyzed using the Kruskal Wallis test. Increased CVD risk percentage was observed in Q4 as compared to patients with leptin levels within Q1 and Q2 in both men and women (Figure 1, *p *< 0.05).

As shown in Table 2, Spearman's rank correlation (*r*_*s*_) analysis of leptin level with cardiometabolic risk factors for men and women was undertaken. In both men and women, leptin levels were positively correlated with BMI, waist circumference, blood pressure, total cholesterol, triglyceride, FPG, uric acid, LDL cholesterol, and percentage of CVD risk, but negatively correlated with HDL cholesterol. In addition, age was positively correlated with leptin levels in women (Table 2).

**Table 2:** ^a^Spearmen's correlation of leptin levels with cardiometabolic risk factors in men and women.

	Men		Women	
	
Cardiometabolic risk factors	*r*_*s*_	*P*-value	*r*_*s*_	*P*-value
Age, y	0.060	0.119	0.161	0.008*
Body mass index, kg/m^2^	0.718	<0.001*	0.629	<0.001*
Waist circumference, cm	0.743	<0.001*	0.502	<0.001*
Tobacco usage (Yes vs. No)	0.012	0.744	-0.062	0.309
Alcohol usage (Yes vs. No)	-0.002	0.948	-0.05	0.409
Systolic blood pressure, mm Hg	0.275	<0.001*	0.199	0.001*
Diastolic blood pressure, mm Hg	0.28	<0.001*	0.195	0.001*
Total cholesterol, mg/dL	0.203	<0.001*	0.185	0.002*
Triglyceride, mg/dL	0.364	<0.001*	0.298	<0.001*
Fasting plasma glucose, mg/dL	0.246	<0.001*	0.243	<0.001*
Uric acid, mg/dL	0.329	<0.001*	0.239	<0.001*
HDL cholesterol, mg/dL	-0.337	<0.001*	-0.227	<0.001*
LDL cholesterol, mg/dL	0.264	<0.001*	0.256	<0.001*
Homocysteine, umol/L	-0.027	0.474	0.059	0.345
CVD risk, %	0.257	<0.001*	0.246	<0.001*

^a ^The coefficient of Spearmen correlation were shown as *r*_*s *_with a P-value.

* P < 0.05.

Univariate linear regression analysis after adjusting for age or multiple variables, including age, tobacco usage, and BMI, to determine the association of leptin levels with cardiometabolic risk factors was assessed (Table 3). After adjusting for age, leptin levels were significantly positively associated with BMI, waist circumference, blood pressure, total cholesterol, triglyceride, uric acid, LDL cholesterol, percentage CVD risk, and metabolic syndrome, but were negatively associated with HDL cholesterol in both men and women. After adjusting for age, BMI, and tobacco usage, leptin levels were positively associated with metabolic syndrome in females (Table 3).

**Table 3:** ^a^Univariate analysis of leptin levels with cardiometabolic risk factors after adjusting for age and tobacco usage in men and women.

	Men				Women			
	
	Age-adjusted		Multivariable-adjusted^b^		Age-adjusted		Multivariable-adjusted^b^	
Cardiometabolic risk factors	B (SE)	*P*-value	B (SE)	*P*-value	B (SE)	*P*-value	B (SE)	*P*-value
Body mass index, kg/m^2^	0.810 (0.033)	< 0.001*	-		1.230 (0.079)	< 0.001*	-	
Tobacco usage (Yes vs. No)	0.342 (0.313)	0.275	-		-1.345 (1.601)	0.402	-	
Alcohol usage (Yes vs. No)	0.174 (0.309)	0.573	0.006 (0.238)	0.978	0.152 (0.805)	0.851	-0.109 (0.608)	0.858
Waist circumference, cm	0.312 (0.013)	<0.001*	0.147 (0.028)	<0.001*	0.394 (0.032)	<0.001*	0.074 (0.050)	0.135
Systolic blood pressure, mm Hg	0.095 (0.012)	<0.001*	0.016 (0.010)	0.103	0.099 (0.026)	<0.001*	-0.038 (0.021)	0.074
Diastolic blood pressure, mm Hg	0.138 (0.017)	<0.001*	0.026 (0.014)	0.074	0.130 (0.038)	0.001*	0.002 (0.029)	0.934
Total cholesterol, mg/dL	0.024 (0.005)	<0.001*	0.008 (0.004)	0.030	0.021 (0.010)	0.043*	0.003 (0.008)	0.678
Triglyceride, mg/dL	0.008 (0.002)	<0.001*	0.002 (0.001)	0.173	0.012 (0.004)	0.006*	-0.003 (0.003)	0.351
Fasting plasma glucose, mg/dL	0.024 (0.010)	0.016*	0.007 (0.007)	0.323	0.024 (0.020)	0.242	-0.024 (0.015)	0.115
Uric acid, mg/dL	0.908 (0.116)	<0.001*	0.199 (0.094)	0.034	1.378 (0.301)	<0.001*	0.282 (0.245)	0.250
HDL cholesterol, mg/dL	-0.079 (0.013)	<0.001*	0.008 (0.011)	0.453	-0.081 (0.024)	0.001*	-0.011 (0.018)	0.542
LDL cholesterol, mg/dL	0.030 (0.005)	<0.001*	0.006 (0.004)	0.115	0.037 (0.011)	0.001*	0.011 (0.008)	0.167
Homocysteine, umol/L	0.060 (0.039)	0.121	0.039 (0.029)	0.175	0.034 (0.192)	0.860	-0.060 (0.140)	0.670
CVD risk, %	0.406 (0.049)	<0.001*	0.096 (0.054)	0.076	0.556 (0.123)	<0.001*	-0.056 (0.105)	0.598
Metabolic syndromes (Yes vs. No)	2.089 (0.325)	<0.001*	0.339 (0.256)	0.186	2.146 (0.685)	0.002*	0.338 (0.520)	0.032*

^a ^The results of univariate analysis were shown as estimated beta (B), standard error of beta (SE), and P-value.

^b^adjusted for age, BMI, and tobacco usage.

* P < 0.05, P-values were derived as for the significance of B from the general linear model analysis.

The correlation between metabolic syndrome and serum leptin quartiles was calculated independent of age, tobacco usage, and BMI in three models using binary logistic regression analysis (Table 4). Specifically, model 1 adjusted for age whereas models 2 and 3 adjusted for age and tobacco usage and age, tobacco usage, and BMI, respectively. In men, compared to the lowest leptin quartile, the odds ratio (OR) and 95% confidence intervals (95% CI) for metabolic syndrome after adjusting for age were 3.28 (2.06 - 5.20) in Q2, 5.12 (3.13 - 8.37) in Q3 and 6.14 (3.70 - 10.19) in Q4 (*p *< 0.001). A similar increase in odds ratio was observed in women (Q4 vs. Q1, 2.94 (1.36 - 6.37), *p *= 0.037). Further adjusting for tobacco usage and BMI revealed increased odds ratio of metabolic syndrome in men only (Q4 vs. Q1, 6.15 (3.71 - 10.21) and 2.66 (1.39 - 5.11), *p *≤ 0.001 for models 2 and 3, respectively); a significantly increased odds ratio was only seen in model 2 for women (Table 4).

**Table 4:** ^a^Odds ratio of metabolic syndromes with leptin levels by sex-specific quartiles.

Leptin levels		**Model 1**^**c**^	**Model 2**^**d**^	**Model 3**^**e**^
**Men**				
Q1	(n = 149)	1.00	1.00	1.00
Q2	(n = 186)	3.28 (2.06 - 5.20)	3.27 (2.06 - 5.19)	2.46 (1.52 - 4.01)
Q3	(n = 181)	5.12 (3.13 - 8.37)	5.11 (3.13 - 8.37)	2.94 (1.67 - 5.17)
Q4	(n = 171)	6.14 (3.70 - 10.19)	6.15 (3.71 - 10.21)	2.66 (1.39 - 5.11)
*P*-value for trend^b^		< 0.001*	< 0.001*	0.001*

**Women**				
Q1	(n = 65)	1.00	1.00	1.00
Q2	(n = 67)	1.27 (0.58 - 2.77)	1.26 (0.57 - 2.75)	1.11 (0.50 - 2.45)
Q3	(n = 68)	1.63 (0.76 - 3.50)	1.61 (0.75 - 3.46)	1.21 (0.54 - 2.71)
Q4	(n = 69)	2.94 (1.36 - 6.37)	2.90 (1.34 - 6.30)	1.55 (0.61 - 3.93)
*P*-value for trend^b^		0.037	0.040	0.816

^a ^The results were represented as estimated Odds ratio (OR) with a 95% confidence interval (95%CI).

^b ^*P*-values for trend were derived from binary logistic regression model analysis.

^c ^Model 1: adjusted for age.

^d ^Model 2: adjusted for age and tobacco usage.

^e ^Model 3: adjusted for age, tobacco usage, and BMI.* P < 0.05.

**Figure 2:**
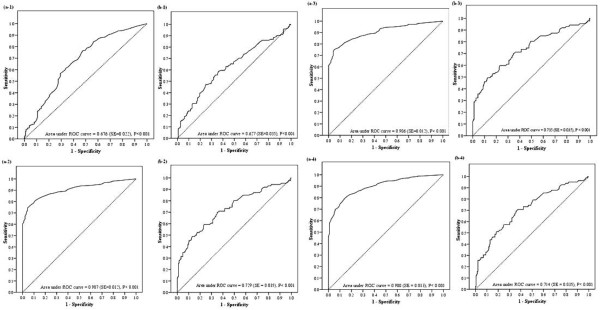
**Receiver Operating Curve (ROC) analyses for leptin levels as a predictor of metabolic syndrome**. ROC analysis was undertaken in (a) men, and (b) women. a-1 and b-1, Leptin vs. Metabolic syndrome without adjustment. a-2 and b-2, age-adjusted. a-3 and b-3, age and tobacco usage-adjusted. a-4 and b-4, age, tobacco usage, and BMI-adjusted. The area under the ROC curves were 0.676 (SE = 0.022), 0.907 (SE = 0.012), 0.906 (SE = 0.012), and 0.900 (SE = 0.013) in men and 0.627 (SE = 0.035), 0.729 (SE = 0.035), 0.735 (SE = 0.035), and 0.714 (SE = 0.035) in women.

The relationship between leptin levels and metabolic syndrome was further explored using ROC analysis to determine the predictive value of leptin levels for metabolic syndrome in men and women (Figure 2). As leptin levels increased, the incidence of metabolic syndrome also significantly increased in both men (0.676 [SE = 0.022]) and women (0.627 [SE = 0.035]); Figure 2a-1 and 2b-1, respectively). The predictive value of leptin levels for metabolic syndrome was not diminished upon adjusting for age (Figure 2a-2 and 2b-2), age and tobacco usage (Figure 2a-3 and 2b-3), and age, tobacco usage, and BMI (Figure 2a-4 and 2b-4) in either sex.

## Discussion

In the present study, serum leptin levels were associated with metabolic syndrome as well as cardiovascular risk in an adult Taiwanese population. Specifically, participants with higher leptin levels had increased metabolic risk factors as compared to those with lower leptin levels. Correlation between leptin levels and metabolic syndrome was observed in both male and female participants. Moreover, serum leptin levels were predicative of metabolic syndrome in both sexes.

Although few, population studies describing the relationship between leptin levels and metabolic syndrome have been reported [7-9]. For example, in obese and nonobese Caucasian children, leptin levels were associated with metabolic syndrome [8]. In the Cyprus Metabolism Prospective Cohort Study, leptin as well as its soluble receptor was not only associated with baseline adiposity and metabolic risk factors, but also predicted adiposity, metabolic syndrome, and glucose levels in eighteen-year-old men [22]. Furthermore, in the Framingham Third Generation Cohort, leptin concentrations were associated with increased odds of metabolic syndrome [23]. Importantly, reductions in leptin levels in obese children (mean age: 14.1 y) predicted short- and long-term reductions in body fat and improved lipid levels and insulin sensitivity, suggesting that children retain leptin sensitivity [24]. Reducing daily caloric intake by 500 kilocalories resulted in a 5-10% decrease in body weight, decreased BMI, and a 25% fall in leptin plasma concentrations [25].

The present study is one of the few studies to analyze the relationship between leptin levels and metabolic syndrome in an Asian population. In nondiabetic Taiwanese adolescents, plasma leptin levels were positively correlated to BMI, waist circumference, waist-to-hip ratio, body fat mass, body fat mass percentage, and triglycerides [7]. Furthermore, serum leptin was associated with metabolic syndrome in obese and nonobese [26] as well as diabetic [27] Korean populations. In addition, in a 3-year follow-up study of Japanese children ages 9-10 years, leptin levels increased in those subjects whose BMI increased during the follow-up period [28].

In the present study, increased odds of metabolic syndrome with increasing leptin levels were observed in both sexes upon adjusting for age and tobacco usage. However, after further adjustment for BMI, increased odds of metabolic syndrome with increased leptin levels were only observed in men. Yun et al. [26] reported that leptin levels were more strongly correlated to risk of metabolic syndrome in men compared to women after controlling for confounding variables. It remains possible that gender differences in body fat distribution or effects of sex steroids may account for this result; another possibility is many Taiwanese women keep their weight on a diet, not performing exercise. That might increase the body fat and the leptin level, but not influents the body weight or BMI. However, further studies are necessary to determine the role in adipose distribution (visceral versus subcutaneous) or reproductive hormones and metabolic syndrome.

Leptin levels were also predictive of metabolic syndrome in both sexes, which is similar to other studies. For example, in adult males participating in the Olivetti Prospective Heart Study, leptin levels were predictor of developing metabolic syndrome at 8-year follow-up [29]. In addition, Franks et al. [30] reported that leptin levels predict worsening of metabolic syndrome over time.

Leptin has a certain degree of influence on appetite, energy consumption, adipose synthesis, and insulin function. Acting as a hormone, leptin has a broad spectrum of biological function; the main effects are the regulation of adipose tissue and body weight [31-34]. First, the inhibition of appetite and the consumption of body energy are achieved through decreased neuropeptide Y (NPY) secretion and hormone secretion of melanocytes, resulting from the combination of leptin and its receptor within the hypothalamus. Second, the increased consumption of energy is achieved by releasing the heat, transformed from the massive storage of energy. The process is due to the increased activities of sympathetic nerves induced by leptin and the activation of the epinephrine receptor on the membranes of the adipose cells. Third, the synthesis of the adipose tissue is directly influenced by leptin, and the resorption can be accelerated. Other researchers suspect that leptin can promote the maturation of adipose cells [35]. Finally, leptin may influence other hormones; insulin can accelerate the secretion of leptin and, inversely, leptin has negative feedback on the synthesis and secretion of insulin [14,15,36-38].

Because leptin influences appetite, energy consumption, adipose synthesis, and insulin function, investigators have explored the use of recombinant leptin as a treatment for obesity. For example, in two severely obese children, a genetic mutation in the leptin coding region was observed [39]. Improvements in fasting serum glucose, insulin levels and glycosylated hemoglobin were observed upon recombinant methionyl human leptin therapy [39]. In addition, leptin therapy corrected the excess appetite and promoted weight loss and puberty initiation in one of the children [40]. However, the single-leptin gene mutation only explains rare cases of human obesity; only 5% of obese patients lack leptin. Most patients are genetically stable and without the single-gene mutation [41]. Despite the lack of leptin mutations, leptin mRNA expression level in the adipose tissue and the serum leptin levels have been positively associated with the degree of obesity [42], suggesting that leptin resistance might be one of the causes for human obesity. However, use of recombinant leptin for obesity has been relatively ineffective.

Obese individuals often have hyperleptinemia, suggesting the presence of leptin resistance which is characterized by the persistence of overweight despite high leptin concentrations. In leptin resistance, the higher leptin concentrations muted the responses to decrease weight [43]. It remains possible that the individuals with upper quartile leptin levels had leptin resistance; however, further studies are necessary to determine if these individuals were leptin resistant and if so, the mechanism by which the leptin resistance manifests (e.g., the presence of leptin neutralizing antibodies or antagonists [44], decreased leptin transport across the blood-brain barrier [45], defective leptin signaling [46], etc.).

This study has some limitations that warrant discussion. Firstly, the retrospective nature of the study prevents determining the direction of causality for the relationship between leptin and metabolic syndrome. Secondly, leptin level is selective item of routine physical examination, usually was chosen by subjects who are aware of overweight or hyperlipidemia. So the prevalence of metabolic syndrome was a litter higher than other studies. Furthermore, because the study population consisted of only Taiwanese adults, the findings may not carry over to other racial or ethnic groups.

In conclusion, serum leptin levels are correlated with CVD risk and metabolic syndrome in Taiwanese adults. Serum leptin levels could also predict metabolic syndrome; therefore, analysis of leptin as part of routine physical examinations may prove beneficial in the early diagnosis of metabolic syndrome.

## Abbreviations

ATP III: Adult Treatment Panel; BMI: Body mass index; CVD: Cardiovascular diseases; FBG: Fasting blood glucose; HDL-C: High-density lipoprotein cholesterol; LDL-C: Low-density lipoprotein cholesterol; OR: Odds ratio; ROC: Received operating curve; NPY: Neuropeptide Y; Q: Quartile; TG: Triglyceride

## Competing interests

The authors declare that they have no competing interests.

## Authors' contributions

WCL and KYH contributed to Study Design; WCL, KYH, YCC and KHW contributed to Conduct/data collection; ICC and SHW contributed to Data Analysis; WCL and KYH contributed to Manuscript Writing. All the authors have read and approved the final version of the manuscript.
